# In Vitro Digestion and Fecal Fermentation of Arecanut Polysaccharides: Effects on Gut Microbiota and Metabolites

**DOI:** 10.3390/foods14172954

**Published:** 2025-08-25

**Authors:** Xiaolong Ji, Ke Jiang, Yuqing Liu, Chenyu Zhao, Jun Du, Liang Chen, Zhigang Zhu, Xiaoqiong Li

**Affiliations:** 1 College of Food and Bioengineering, Zhengzhou University of Light Industry, Zhengzhou 450001, China; xiaolongjiytu@163.com (X.J.); jk15993393809@163.com (K.J.); 14749600418@163.com (Y.L.); 18837557582@163.com (C.Z.); 2 Institute of Food Science, Zhejiang Academy of Agricultural Sciences, Hangzhou 310021, China; 3 Nutrilite Health Institute, Amway (Shanghai) Innovation & Science Co., Ltd., Shanghai 201203, China; eric.du@amway.com (J.D.); clark.chen@amway.com (L.C.)

**Keywords:** arecanut, polysaccharides, in vitro fermentation, gut microbiota, metabolites

## Abstract

Recent studies have increasingly emphasized the regulatory potential of plant-derived polysaccharides on gut microbial composition and metabolic function. Despite this growing interest, investigations focusing specifically on the simulated digestion and fermentation properties of arecanut polysaccharide (PAP1b) remain limited. In this work, we employed the standardized INFOGEST 2.0 protocol to mimic the oral, gastric, and intestinal digestion of PAP1b, followed by 48 h anaerobic fermentation using pooled human fecal samples from healthy adult donors. PAP1b treatment led to a progressive decrease in pH and a substantial elevation in SCFAs levels, notably acetic, propionic, and butyric acids. Simultaneously, PAP1b significantly promoted the growth of SCFA-producing microbial taxa, particularly members of the *Firmicutes* phylum such as *Lachnospiraceae*, *Lachnoclostridium*, *Bilophila*, and *Phascolarctobacterium*, while markedly suppressing *Bacteroidota* populations. Metabolomic analysis further indicated that PAP1b intake enhanced bile acid metabolism, suggesting its potential as a prebiotic candidate for improving intestinal health.

## 1. Introduction

The arecanut (*Areca catechu* L.), a member of the *Arecaceae* family, ranks among the most economically significant crops cultivated in tropical regions of Asia and certain areas of Africa [1]. Research indicates that arecanut contains a diverse array of bioactive compounds, including flavonoids, tannins, alkaloids, triterpenoids, steroids, fatty acids, and polysaccharides, etc. Due to its rich phytochemical composition, arecanut holds substantial commercial and nutritional importance [2]. Arecanut polysaccharide (AP) is a macromolecular compound extracted from arecanut nut fruits, seeds, or processed residues, and belongs to a kind of plant polysaccharide with various biological activities [3]. AP is the primary bioactive component, which has immunomodulation, antioxidant, anti-inflammatory regulating gut microbiota effects, and our previous study successfully extracted and isolated a neutral polysaccharide (PAP1b) through aqueous alcoholic precipitation and column chromatography [4]. Comprehensive structural analysis revealed that PAP1b possessed a heteropolymeric architecture dominated by a backbone of alternating →6)-*β*-D-mannopyranosyl-(1→ and →4)-α-D-galactopyranosyl-(1→ glycosidic linkages. Branching occurred at periodic intervals via →3,6)-*β*-D-mannopyranosyl-(1→ junction residues, with terminal non-reducing ends predominantly occupied by *β*-D-mannopyranose units linked through (1→) bonds. Accumulating evidence suggests that plant polysaccharides could modulate gut microbial ecology through enhancing the abundance of beneficial bacteria and influencing host metabolic pathways [5,6]. To date, the in vitro fermentation characteristics of arecanut polysaccharides (AP) and their subsequent effects on gut microbial ecology and metabolic pathways remain largely unexplored, with the exception of a single study by Zhang et al. [3]. This notable paucity of research underscores the need for systematic investigations into the potential bioactivity of AP, particularly its capacity to modulate commensal microbiota and associated metabolic functions. Based on existing evidence linking structural features of plant polysaccharides to prebiotic activity, we postulated that AP might exert clinically relevant bioeffects through targeted microbial interactions and downstream metabolic regulation.

Digestive and colonic fermentation simulations in vitro have become widely adopted tools for investigating the functional properties of polysaccharides derived from plant sources [7,8]. These models could effectively simulate the digestive and fermentative processes within the human body, enabling the assessment of plant polysaccharide degradation during digestion and their subsequent impact on intestinal microbiota [9]. While in vitro fermentation systems could also offer useful information on microbial activity under standardized conditions, they cannot entirely mimic the intricate interactions between the host and gut microbiota observed in vivo. Mounting evidence suggests that the gut microbiome plays a central role in epithelial integrity, immune modulation, and metabolic regulation [10]. These commensal microorganisms form a complex symbiotic relationship with their host, contributing to nutrient metabolism, pathogen exclusion, and immune system maturation. Of particular clinical relevance is the well-documented association between gut microbial dysbiosis and the pathogenesis of various metabolic conditions. Epidemiological and mechanistic studies have consistently demonstrated that alterations in microbial composition and function are intimately linked to the development and progression of obesity, insulin resistance, type II diabetes mellitus, and atherosclerotic cardiovascular diseases [11,12]. Among the strategies to modulate gut microbiota composition, dietary interventions are particularly effective. Polysaccharides, due to their resistance to digestion by the host gastrointestinal enzymes, could reach the colon intact, where they are fermented through gut microbes to produce beneficial metabolites such as short-chain fatty acids (SCFAs), exhibiting promising prebiotic potential [13,14]. Human fecal in vitro fermentation systems could offer a reliable and ethical approach to mimic colonic microbial fermentation, facilitating the assessment of plant polysaccharide degradability and associated changes in microbiota composition and metabolite profiles [15]. In vitro experimental models serve as a valuable platform for evaluating the prebiotic potential of AP, particularly their selective modulatory effects on gut microbial populations. These controlled systems could enable precise characterization of AP’s ability to promote the proliferation of beneficial commensal bacteria while simultaneously inhibiting the growth of opportunistic pathogens. Such differential microbial regulation represents a promising therapeutic strategy for restoring ecological balance within the intestinal microenvironment, as demonstrated in prior investigations [3].

This study was designed to address two key research questions: characterize the digestive behavior and structural integrity of PAP1b under simulated gastrointestinal conditions, and systematically investigate its impact on microbial community dynamics, short-chain fatty acid (SCFA) production, and metabolic profile alterations during colonic fermentation. This study could present a systematic evaluation of PAP1b’s functional properties, aiming to support its potential development as a prebiotic through assessment of its fermentation characteristics, influence on microbial communities, and metabolite profiles in a simulated digestive environment.

## 2. Materials and Methods

### 2.1. Materials and Chemicals

The present investigation utilized premium-grade arecanut (*Areca catechu* L.) specimens procured from Hunan Kouweiwang Co., Ltd. (Yiyang, China), a certified supplier of botanical materials. For the enzymatic digestion protocols, we employed high-purity biochemical reagents from Sigma-Aldrich (St. Louis, MO, USA), specifically gastric pepsin, pancreatic α-amylase, and pancreatin. SCFAs, including acetic, propionic, n-butyric, i-butyric, n-valeric, i-valeric, and 2-ethylbutyric acids, were obtained from Aladdin Chemical Reagent (standards for GC, purity ≥ 99.5%, Shanghai, China).

### 2.2. Extraction and Purification of PAP1b

The isolation and purification of arecanut polysaccharide (PAP1b) was conducted according to an optimized protocol adapted from established methodologies [4]. The extraction process commenced with defatted arecanut powder (particle size < 150 μm) undergoing ultrasonic-assisted extraction (40 kHz, 500 W) in hot water at 80 °C for 90 min, maintaining a precise solid-to-liquid ratio of 1:25 (*w*/*v*). The resulting supernatant was concentrated under the reduced pressure at 60 °C, followed by graded ethanol precipitation (final concentration 80% *v*/*v*) at 4 °C for 12 h to obtain crude arecanut polysaccharide (AP). The AP fraction was then subsequently dialyzed (MWCO 3.5 kDa) against distilled water for 48 h with frequent water changes, then lyophilized. For primary purification, the AP solution was chromatographed on a DEAE-Sepharose Fast Flow column (2.6 × 100 cm) pre-equilibrated with the same buffer. Elution was performed with a linear NaCl gradient (0–0.3 M) at 0.5 mL/min, with 5 mL fractions collected and analyzed for carbohydrate content through the phenol–sulfuric acid method (λ = 490 nm). The major polysaccharide peak (eluted with distilled water) was concentrated and further purified by size-exclusion chromatography on a Sephacryl S-100 HR column (2.6 × 100 cm) using ultrapure water as the mobile phase (0.3 mL/min). The purified PAP1b fraction (elution volume 120–150 mL) was lyophilized and stored in a vacuum desiccator at 4 °C until further analysis.

### 2.3. In Vitro Digestion of PAP1b

#### 2.3.1. Simulated Digestive Solution Preparation

The preparation of simulated digestive fluids required the following solutions to be accurately prepared at specified molar concentrations: 0.3 M calcium chloride dihydrate (CaCl_2_·2H_2_O), 0.5 M potassium chloride (KCl), 0.5 M potassium dihydrogen phosphate (KH_2_PO_4_), 1 M sodium bicarbonate (NaHCO_3_), 2 M sodium chloride (NaCl), 0.15 M magnesium chloride hexahydrate (MgCl_2_·6H_2_O), 0.5 M ammonium carbonate ((NH_4_)_2_CO_3_), 5 M sodium hydroxide (NaOH), and 1 M hydrochloric acid (HCl). A 400 mL volume of concentrated (1.25×) simulated digestive fluid was prepared and could be stored at −20 °C for extended periods up to one year. Notably, to prevent precipitation during storage, the calcium chloride dihydrate component was excluded from the stock solution and instead added immediately prior to conducting digestion experiments. This precaution maintained solution stability while ensuring all required ionic components were present during actual experimental procedures. Three simulated digestion solutions, namely simulated salivary fluid (SSF), simulated gastric fluid (SGF), and simulated intestinal fluid (SIF), were prepared in accordance with the standards outlined in INFOGEST 2.0 [16]. The prepared digestion solutions served as the basis for formulating the following enzyme solutions: salivary α-amylase at 10 mg/mL concentration for carbohydrate breakdown, porcine pepsin solution at 20 mg/mL for protein digestion, pancreatic enzyme mixture at 133.3 mg/mL concentration containing various digestive enzymes, and bile salt solution at 200 mg/mL for lipid emulsification. Special attention was given to the bile solution preparation, which required incubation with a temperature-controlled shaker at 37 ± 0.5 °C for a minimum of 30 min to achieve complete solubilization of bile components. All enzyme solutions were aliquoted and stored at −4 ± 1 °C to maintain stability, with thorough pre-warming to physiological temperature (37 °C) in a calibrated water bath for 15 min immediately before experimental use to ensure proper enzymatic activity [17].

#### 2.3.2. Oral Digestion

The salivary digestion of PAP1b was performed using a modified Zhu et al. [18] protocol. Briefly, 500 mg PAP1b was dissolved in 2 mL ultrapure water (1:4 *w*/*v* ratio). A 1.25× concentrated simulated salivary fluid (SSF) and α-amylase (Type XIII-A, ≥300 U/mg) were pre-warmed to 37 °C. The reaction mixture contained 1.6 mL SSF, 2 mL PAP1b solution, 10 μL 0.75 M CaCl_2_·2H_2_O, and 300 μL α-amylase (50 U/mL final concentration), adjusted to 4 mL total volume with water to achieve 1× SSF concentration. After vortex mixing, the sample was incubated at 37 °C for 2 min thorough shaking (100 rpm), then immediately cooled on ice and centrifuged (10,000× *g*, 10 min, 4 °C) to terminate the reaction.

#### 2.3.3. Gastric Digestion

Based on the approach described by Di et al. [19], the method was modified to suit the experimental conditions of this study. We slowly added 3.2 mL of 1.25 times the concentration of SGF to 4 mL of the oral chyme mixture at the end of the oral phase, and then carefully adjusted the mixture of pH to 3.0 using a 1 M HCl solution to ensure an accurate digestive environment. Next, we added 2 µL of CaCl_2_(H_2_O)_2_ solution and 266.8 µL of porcine pepsin solution that had been preheated to the appropriate temperature to the mixture and stirred well. In order to adjust the concentration of the mixture to 1× the concentration of SGF, we supplemented the mixture with a moderate amount of deionized water. Upon introducing pepsin, the 8 mL digestion mixture was immediately transferred to a 37 °C thermostatic shaker for continuous agitation and incubation over a two-hour period, ensuring complete gastric phase digestion.

#### 2.3.4. Small Intestinal Digestion

A previously established method by Lu et al. [20] was employed with necessary adaptations for this investigation. We transferred 8 mL of the gastric coeliac mixture to a new container at the end of the gastric phase and added 3.2 mL of a 1.25-fold concentration of SIF slowly, and then carefully adjusted the mixture of pH to 7.0 with a 5 M NaOH solution to mimic the physiological environment of the intestine. Following this step, 1.2 mL of bile solution was introduced into the mixture and thoroughly stirred to achieve homogeneous integration with the coeliac components. Subsequently, 16 µL of CaCl_2_·2H_2_O solution with 2 mL of pancreatic enzyme was incorporated, followed by vigorous mixing to ensure uniform dispersion of the enzyme throughout the digestive medium. We again verified the mixture of pH and fine-tuned it, if necessary, to maintain it at 7.0. To adjust the concentration of the mixture to 1× the concentration of SIF, we supplemented the mixture with a moderate amount of deionized water. From the moment trypsin was added, we placed this 16 mL of digestion mixture in a thermostatic shaker at 37 °C for thorough mixing and incubation for a duration of 2 h to ensure that the entero-digestive phase was adequately carried out. At the end of digestion, we inactivated the mixture using a 70 °C water bath for 5 min to stop the digestion reaction. Subsequently, we stored the digested samples for subsequent dialysis and concentration treatment for fermentation experiments. A sample aliquot from the digested mixture was subjected to centrifugation at 8500 rpm for 12 min under 4 °C conditions to isolate the supernatant. Following separation, the supernatant fraction was preserved at −20 °C for further analysis.

### 2.4. In Vitro Fermentation of PAP1b

#### 2.4.1. Design for Fecal Fermentation

Fresh fecal samples were collected from 6 healthy donors (3 males, 3 females; aged 20–30 years) who had abstained from antibiotic use for ≥3 months. After obtaining written informed consent and approval from the Zhejiang Academy of Agricultural Sciences Ethics Committee (No. 2023-025), samples were processed within 2 h of collection. Each specimen (0.6–0.7 g) was homogenized with 6–7 mL of sterile physiological saline (0.9% NaCl) to prepare a 10% (*w*/*v*) suspension. The mixture was vortexed for 3 min, filtered through four layers of sterile gauze to remove particulate matter, and centrifuged (500× *g*, 5 min) to obtain a clear supernatant [21]. The resulting fecal inoculum was immediately used for fermentation studies or stored anaerobically at −80 °C for ≤2 weeks.

After mixing the filtrate with the PAP1b-supplemented medium, the mixture was incubated anaerobically at 37 °C. In order to verify the accuracy of the experimental results, we set up two control groups: a blank control group (CK, negative control), which consisted of medium, and a positive control group (INU), which consisted of medium with added inulin. Each sample was processed separately to serve as a biological replicate. For each donor sample, fermentation was conducted in triplicate under identical conditions to ensure technical reproducibility. Throughout the incubation period, specimens were obtained at five key intervals (0, 6, 12, 24, and 48 h) to monitor dynamic changes. Following collection, each sample underwent centrifugation at 8000× *g* for 10 min, effectively partitioning the liquid supernatant from the solid fecal residue. Both fractions were immediately flash-frozen and preserved at −80 °C to ensure molecular stability for future analytical procedures.

#### 2.4.2. pH Determination

During the fermentation process, we strictly followed the experimentally designed time points for sample collection. Specifically, we used a 1 mL syringe to accurately draw 0.5 mL of fermentation samples at the baseline time point (i.e., 0 h), as well as at each of the five different subsequent key moments: 6 h, 12 h, 24 h, and 48 h. To ensure data accuracy, a pH meter was immediately used to measure the pH of each sample collected, and the measurements were carefully recorded for subsequent data analysis and interpretation of the experimental results [22].

#### 2.4.3. Determination of SCFAs by GC

The calibration standards were prepared through a two-step process to ensure accuracy. First, precise 200 mL aliquots of acetic acid and propionic acid were individually measured and transferred to separate sterile containers. These primary solutions were then quantitatively blended with a pre-optimized mixture of four additional short-chain fatty acids (isobutyric acid, n-butyric acid, isovaleric acid, and n-pentanoic acid) to yield the final calibration standard. Specifically, we performed this operation in a 10-milliliter volumetric flask, adding first the acetic acid and then the propionic acid to a solution that also contained several other short-chain fatty acids. Next, we added 1 mL of ultrapure water to another 10 mL volumetric flask for each short-chain fatty acid (including the previously added acetic and propionic acids) to ensure homogeneity and stability of the solution. The fermented mixture was transferred to 15 mL centrifuge tubes and centrifuged at 8000 rpm for 10 min. After centrifugation, the supernatant was carefully collected into new sterile tubes and promptly frozen for subsequent SCFA analysis. Prior to SCFA quantification, the preserved acidic fermentation samples underwent additional centrifugation at 12,000 rpm for 10 min to ensure complete clarification. For chromatographic analysis, 2 µL of the processed supernatant was injected into a DB-FFAP capillary column (60 m × 0.25 mm × 0.5 µm, Agilent Technologies were sourced from Santa Clara, CA, USA). The gas chromatography analysis was performed using a Shimadzu QP2010 system with helium carrier gas maintained at a constant flow rate of 0.7 mL/min throughout the analysis. The protocol initiated with a temperature stabilization phase at 50 °C lasting for 1 min, followed by a temperature gradient increasing at 15 °C per minute up to 240 °C, and concluding with a 10 min isothermal period at the final temperature to ensure complete elution of all short-chain fatty acids. This analytical approach provided reliable quantification of SCFA concentrations in the samples [23].

#### 2.4.4. Determination of Gas Production During In Vitro Fermentation

In order to detect the gases produced more accurately during the fermentation process, we opened the detection port of the gas composition analyzer and simultaneously enabled the inlet valve connected to the air source, and thoroughly purged and cleaned the inside of the instrument to ensure the purity of the measurement environment and the data accuracy. After cleaning, we carefully placed the vials containing the fermentation broth in the designated slots of the instrument. Then, we started the analyzer to measure the gas composition of the vials, covering all the key indicators such as carbon dioxide (CO_2_), methane (CH_4_), hydrogen (H_2_), and hydrogen sulfide (H_2_S), in order to obtain detailed and accurate information on the gas production and changes during the fermentation process [24].

### 2.5. DNA Extraction and 16S rRNA Gene Sequencing

After a 48 h fermentation process, the sediment samples obtained were carefully transferred to MajorBio (Shanghai, China) for detailed 16S rRNA gene sequencing. Cecal contents were aseptically collected by extrusion from the cecum. The bacterial 16S rRNA gene (V3-V4 regions) was amplified using the primers 338F (5′-ACTCCTACGGGAGGCAGCA-3′) and 806R (5′-GGACTACHVGGGTWTCTAAT-3′) on an ABI GeneAmp^®^ 9700 thermal cycler (Applied Biosystems, Foster City, CA, USA) [25]. PCR products were subsequently purified with a DNA gel extraction kit (Axygen Biosciences, Union City, CA, USA) and quantified using the QuantiFluor-ST fluorometer. Sequencing was performed using the Illumina MiSeq platform, following the standard protocols provided by Majorbio Bio-Pharm Technology Co., Ltd., Shanghai China. The raw sequence reads have been submitted to the NCBI Sequence Read Archive (SRA) under accession number PRINA935523. After sequencing, paired-end reads were demultiplexed and subjected to quality filtering. High-quality overlapping sequences were merged to yield clean tags. Denoising and amplicon sequence variant (ASV) inference were conducted using the DADA2 pipeline to produce representative sequences along with their abundance profiles. Taxonomic classification was performed against the SILVA 138/16S rRNA reference database using a confidence threshold of 0.7 [26]. This approach yielded high-resolution microbial community profiles, enabling in-depth taxonomic analysis.

### 2.6. Analysis of Intestinal Microbial Metabolites

Following the 48 h in vitro fermentation, 400 μL of supernatant was precisely aliquoted from the fermented medium and combined with 1.2 mL of a chilled methanol: acetonitrile (1:1 *v*/*v*) solution for metabolite extraction. The resulting mixture was subjected to intensive vortexing for 30 s to achieve thorough mixing, followed by sonication at 40 kHz for 30 min to maximize compound solubilization. To ensure effective protein precipitation and phase separation, the samples were then incubated at −20 °C for 3 h. Subsequent centrifugation at 10,000× *g* for 10 min (4 °C) yielded a particulate-free supernatant, from which 750 μL was carefully pipetted into vials for immediate UPLC-MS/MS analysis, with a separate 50 μL portion retained for system suitability testing. Chromatographic analysis was performed using a thermostat (40 °C) Waters ACQUITY UPLC BEH C18 column (2.1 × 100 mm, 1.7 μm particle size). The gradient elution employed a binary solvent system: (A) 0.1% formic acid in 2% acetonitrile and (B) 0.1% formic acid in 98% acetonitrile, delivered isocratically at 0.4 mL/min. A 5.0 μL injection volume was selected to balance detection sensitivity and chromatographic resolution, as previously optimized [8]. Mass spectrometric detection parameters were tuned to ensure comprehensive coverage of the extracted metabolite profile.

### 2.7. Statistical Analysis

All data are presented as mean ± standard deviation (SD). Statistical differences among groups were assessed using one-way ANOVA, followed by Duncan’s multiple range test for post hoc comparisons when appropriate. Analyses were performed with SPSS Statistics version 27.0 (IBM, Armonk, NY, USA), and statistical significance was defined as *p* < 0.05. Data visualization and plotting were conducted using Origin 2021 (OriginLab, Northampton, MA, USA).

## 3. Results and Discussion

### 3.1. Impacts of PAP1b on pH and SCFAs in In Vitro Fermentation

**Figure 1:**
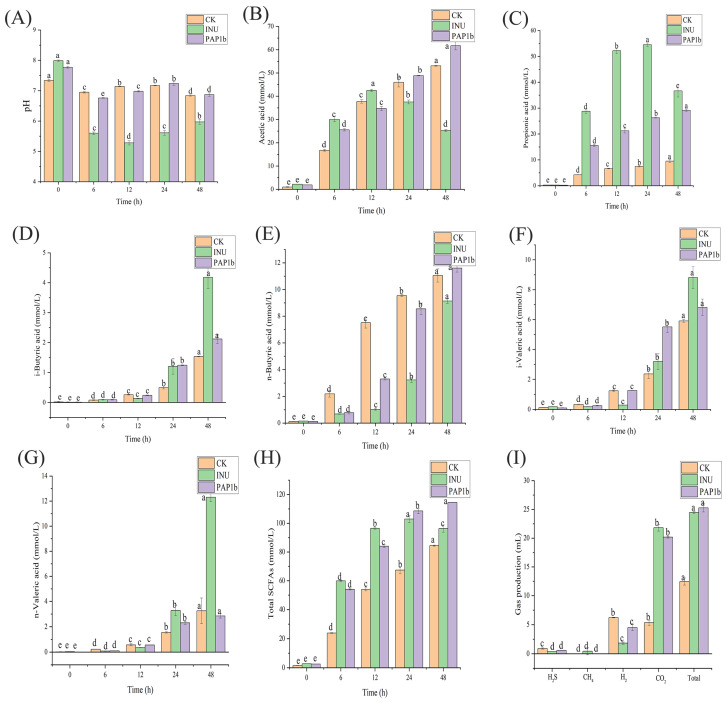
Changes in pH, SCFAs, and gas production during fermentation. (**A**) Variations of pH, (**B**) acetic, (**C**) propionic, (**D**) i-butyric, (**E**) n-butyric, (**F**) i-valeric, (**G**) n-valeric, (**H**) total SCFAs, and (**I**) gas production. Different lowercase letters in the same column indicate a different significance between treatments (*p* < 0.05).

The pH value of microbial environment plays a crucial role in the fermentation process, reflecting the decomposition and utilization of plant polysaccharides by the microbial community [27]. The activity of gut bacteria under acidic conditions is particularly beneficial for host health [28]. As shown in Figure 1A, the pH value of the INU group showed the most significant decrease (*p* < 0.05), from 7.99 to 5.60 after 6 h fermentation, while the pH value of AP1b group also decreased from 7.77 to 6.76, although the decrease was slightly smaller. It may be due to the fact that inulin was soluble and PAP1b had a complex branching structure, which was in agreement with Zhang’s results [29].

The decrease in pH value is likely due to the conversion of plant polysaccharides into organic acids during intestinal fermentation. And this change in pH value, in turn, could have a significant impact on the health of the intestinal environment [30,31]. The intestine could maintain a more acidic environment by adding PAP1b, which is conducive to the growth of beneficial bacteria and also may affect the proliferation of certain specific microbial communities. Therefore, this demonstrates the effectiveness of PAP1b as a potential prebiotic in maintaining intestinal health [32]. The pH change in the microbial environment is a key indicator of the fermentation process, which reflects the activity of microorganisms and their utilization of plant polysaccharides [33]. The addition of PAP1b is beneficial for maintaining the acidic environment of the intestine, thereby promoting intestinal health.

SCFAs are key end products engendered by gut microbiota through endogenous enzyme metabolism encoded by them. These acids are mainly secreted by epithelial cells of the colon mucosa and serve as important components of plant polysaccharide fermentation processes [34]. As illustrated in Figure 1B–H, all experimental groups demonstrated a progressive accumulation of SCFAs throughout the fermentation period, with this metabolic activity being principally attributed to microbial carbohydrate metabolism. The PAP1b treatment group exhibited particularly pronounced SCFA production, with its total SCFA concentration rising dramatically from an initial 2.36 ± 0.02 mmol/L to a final 114.29 ± 1.28 mmol/L after 48 h of fermentation. This final concentration represented a statistically significant increase (*p* < 0.05), compared with both the CK control group (84.38 ± 0.37 mmol/L from a baseline of 1.53 ± 0.11 mmol/L) and the INU group (96.33 ± 2.51 mmol/L from a starting concentration of 2.80 ± 0.04 mmol/L), demonstrating PAP1b’s superior fermentability.

Further analysis revealed that acetic acid, propionic acid, and butyric acid were the main SCFAs produced by the gut microbiota metabolism of PAP1b. After 48 h of fermentation, the concentrations of these three acids reached 61.71 ± 1.78 mmol/L, 29.18 ± 0.43 mmol/L, and 11.61 mmol/L, respectively. These SCFAs have multiple benefits for host health. Acetic acid could regulate insulin signaling pathway and adipocyte synthesis in adipose tissue, providing a new way to prevent type 2 diabetes and obesity [35,36]. Propionates also could promote the proliferation of intestinal epithelial cells, enhance insulin sensitivity, and regulate metabolic homeostasis in the body, while also having a certain impact on cholesterol synthesis; butyric acid, as an energy source for colonic epithelium, has a positive anti-inflammatory regulatory effect [37]. In conclusion, PAP1b was effectively fermented by gut microbiota, resulting in the production of significant levels of SCFAs, which were closely associated with various aspects of intestinal and metabolic health, indicating its potential as a beneficial dietary component.

An interesting observation emerged regarding the INU group’s fermentation profile, where a marked decline in both acetic and propionic acid concentrations became evident during the latter fermentation phase (24–48 h). This temporal reduction potentially reflects selective substrate utilization by specific bacterial populations, suggesting these microbial communities might have preferentially metabolized these SCFAs or their precursors as fermentation progressed. Some studies have found that some bacteria are able to convert lactic acid into butyric acid [38]. Jujube polysaccharides could modulate gut microbiota during in vitro fermentation, promoting the production of SCFAs such as acetic, propionic, and butyric acids, which benefit intestinal health [8]. In addition, gut microbiota could use hexose and pentose as energy sources to produce acetate, propionate, and butyrate. PAP1b has great potential to generate more SCFAs that are beneficial to host health. These SCFAs produced by PAP1b play important roles in regulating body metabolism, enhancing insulin sensitivity, and preventing diseases.

### 3.2. Impacts of PAP1b on Gas Volume in In Vitro Fermentation

Gas production serves as a key indicator of fermentable carbohydrate availability and the extent of microbial fermentation, reflecting the efficiency of microbial nutrient utilization within the substrate [39,40]. As shown in Figure 1I, the total gas production in the PAP1b group (25.27 ± 0.71 mL) exhibited an increasing trend compared with the CK group, and both were higher compared with CK (12.45 ± 0.62 mL). In the in vitro fermentation experiments, the CK group showed the highest H_2_ production (6.21 ± 0.06 mL). This value was significantly higher than that of the PAP1b group (4.50 ± 0.54 mL) and the INU group (1.83 ± 0.19 mL). The lower gas production in the PAP1b and INU groups might suggest that partially undigested polysaccharides were delivered to the distal colon, where they served as fermentation substrates for specific microbial populations. During this process, hydrogen, as an intermediate metabolite of polysaccharide catabolism, may be further competitively consumed by hydrogenophilic microorganisms [41]. This mechanism might have resulted in lower net hydrogen accumulation in the INU and PAP1b groups with exogenous polysaccharide addition. In contrast, the CK group, due to the lack of sufficient amount of exogenous substrate to drive the metabolic activities of the hydrogenotrophic bacteria, might be more inclined to rapidly release hydrogen through endogenous fermentation, while the subsequent hydrogen consumption process failed to proceed adequately due to substrate limitation.

Similarly, H_2_S showed a similar trend, i.e., it was higher in the CK group (0.90 ± 0.11 mL) than in the PAP1b group (0.59 ± 0.01 mL) and the INU group (0.37 ± 0.02 mL). This phenomenon might be attributed to the lack of exogenous plant polysaccharide substrates in the CK group, leading to the preferential fermentation of endogenous substrates, whose metabolic pathways tend to rapidly release hydrogen (H_2_), providing sufficient substrates for hydrogenophilic microorganisms such as sulfate-reducing bacteria (SRB), which drive the synthesis of H_2_S [42]. In contrast, the addition of exogenous polysaccharides (e.g., INU group and PAP1b group) could lead to a decrease in H_2_S production by promoting the activity of other hydrogenotrophic bacteria that form a competitive consumption of H_2_ with SRB. However, CO_2_ showed an opposite trend to the first two gases, and was significantly lower in the CK group (5.34 ± 0.45 mL) than in the PAP1b (20.18 ± 0.17 mL) and INU (21.81 ± 0.58 mL) groups. It may be due to exogenous polysaccharides (e.g., INU and PAP1b) being used as the main substrates for microbial fermentation, which were broken down to pyruvate through the glycolytic pathway, pyruvate was oxidized and decarboxylated to generate acetyl coenzyme A catalyzed by the dehydrogenase complex, and some of the microorganisms may have oxidized the acetyl coenzyme A further through the TCA cycle, which significantly increased the amount of CO_2_ production [43].

In conclusion, the opposite trend of CO_2_ versus H_2_ and H_2_S reflected the global regulation of microbial metabolic networks by exogenous PAP1b: exogenous substrates significantly could enhance CO_2_ production by driving efficient oxidative metabolism and enrichment of CO_2_-producing functional flora, while inhibiting the accumulation of reducing gases (H_2_, H_2_S) through competitive consumption of hydrotropic bacteria.

### 3.3. Impacts of PAP1b on Microbial Communities

#### 3.3.1. Impacts of PAP1b on Microbial Diversity

**Figure 2:**
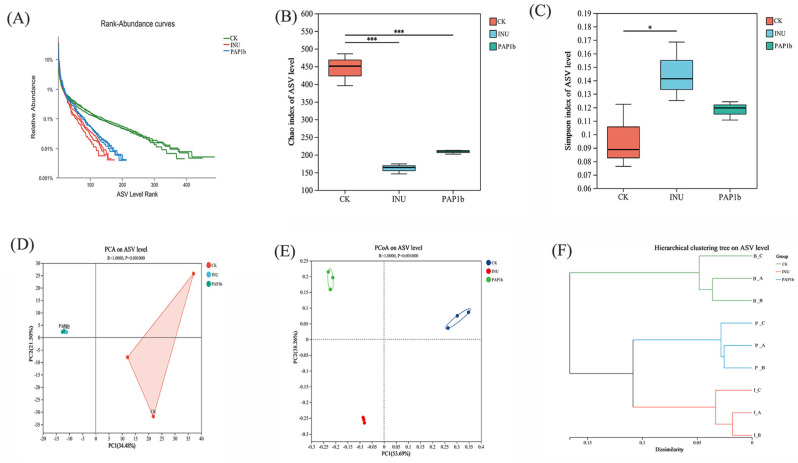
(**A**) Rank abundance curves, (**B**) Chao indexes, (**C**) Simpson indexes, (**D**) PCA analysis, (**E**) PCoA analysis, and (**F**) hierarchical clustering tree. “*” and “***” indicate levels of statistical significance for the comparisons among groups. “*” means *p* < 0.05 (significant difference).“***” means *p* < 0.001 (extremely significant difference).

The effect of PAP1b on the diversity and abundance of in vitro fermented gut microorganisms was explored by 16S rRNA gene sequencing. Species accumulation curves are a powerful tool for assessing the species richness and evenness of samples. Rank abundance curves (Figure 2A) were not only wide but also quite smooth, which suggested a relatively even distribution of species. In addition, the rarefaction curves tended to plateau with increasing sample size, indicating that the sequencing depth was sufficient in the PAP1b, INU, and CK groups to meet basic experimental requirements.

In order to gain a deeper understanding of the diversity of microbial communities, alpha diversity indices were used for evaluation, including Chao index (measuring community richness), Shannon index (reflecting community diversity), and Simpson index (reflecting sample complexity) [44]. As illustrated in Figure 2B, the alpha diversity index was significantly lower (*p* < 0.05) in the PAP1b group compared with the CK group. This finding implied that the addition of PAP1b might have promoted the growth of some specific bacteria, but at the same time reduced the overall abundance and diversity of gut microbiota. This result was in line with previous findings on jujube polysaccharide and *Siraitia grosvenorii* polysaccharide [8,41]. Notably, the Simpson’s index of the PAP1b group was not significantly different from that of the CK group (Figure 2C), suggesting that PAP1b may have driven the metabolic changes through the enrichment of specific functional bacteria, but these functional bacteria may have originally belonged to the dominant taxa in the community. As a result, the relative abundance of dominant bacteria did not fluctuate drastically despite significant changes in the production of metabolic end-products, resulting in a stable diversity index [45]. The stability of the Simpson’s index suggested that the metabolic effect of PAP1b was more likely to be realized through the adjustment of metabolic activity of the functional groups of bacteria or the mechanism of functional redundancy rather than an overall remodeling of the community structure.

To determine whether PAP1b affected microbial diversity, beta diversity analysis was performed. As shown in Figure 2D, PCA1 and PCA2 accounted for 34.48% and 21.50% of the variance, respectively, whereas PCoA1 and PCoA2 explained 53.69% and 38.2% of the variance (Figure 2E), indicating that the reproducibility and similarity among PAP1b differed significantly from that of the CK group. The intestinal flora of the PAP1b group were significantly different from that of the CK group. In addition, the results of the Bray–Curtis cluster analysis supported the findings of the principal component analysis (Figure 2F), further confirming that PAP1b drives functional and compositional differentiation of gut microbial communities by selectively enriching or inhibiting specific functional flora.

In conclusion, 16S rRNA gene sequencing was used to investigate the impact of PAP1b on the composition and diversity of gut microbiota during in vitro fermentation. PAP1b supplementation appeared to promote the growth of certain bacterial taxa while reducing overall microbial abundance and diversity. These results could offer insights into how PAP1b modulates gut microbial communities under controlled conditions.

#### 3.3.2. Impacts of PAP1b on Microbial Component

**Figure 3:**
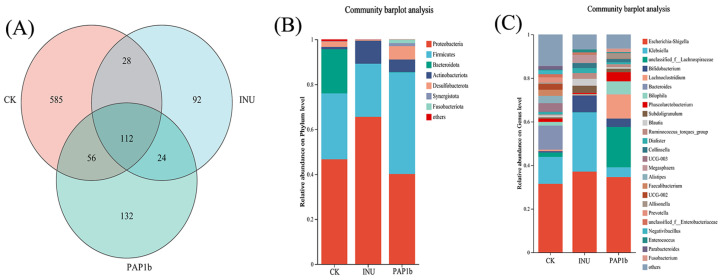
(**A**) Venn diagram of gut microbiota; the composition of gut microbiota at (**B**) phylum level and (**C**) genus level.

The Venn diagram is an effective tool for comparing similarities and differences between different groups. As shown in Figure 3A, the number of ASVs (amplicon sequence variants) common to the three groups was 112, which represented approximately 10.88% of the total number of ASVs. The remaining 89.12% represented ASVs that differed among the groups, with 92 unique ASVs in the INU group and 132 unique ASVs in the PAP1b group. These data revealed that the PAP1b group could show a large variation in the gut microbial species and a slightly significant decrease in the gut microbial species relative to the other groups.

At the phylum level (Figure 3B), *Firmicutes*, *Bacteroidota*, and *Actinobacteria* were the predominant taxa across all groups. Notably, the relative abundance of the *Firmicutes* phylum increased in the PAP1b group compared with the CK and INU groups, while the relative abundance of the *Bacteroidota* phylum decreased significantly. Certain *Firmicutes* members could ferment indigestible dietary polysaccharides, and these bacteria played a positive role in improving intestinal function during the production of butyric acid. Certain bacteria in the thick-walled phylum not only utilized indigestible dietary polysaccharides through fermentation but also promoted intestinal health [46]. It was shown that PAP1b treatment might cause an increase in the F/B (*Firmicutes* to *Bacteroidota* phylum) ratio, which might be related to the lower pH environment produced during fermentation. Because numerous species of the genus Bacteroidetes showed intolerance to low pH environments, they had difficulty surviving in such acidic conditions. On the other hand, the relative abundance of *Actinobacteriota* spp. increased in both PAP1b and INU groups, and the change could be largely attributed to the increase in the number of *Bifidobacterium* spp., which have been recognized as key probiotics in the human gut [35].

Analyses at the genus level, as shown in Figure 3C, revealed specific effects of the PAP1b intervention on the composition of gut microbes. The relative abundance of *Lachnospiraceae*, *Lachnoclostridium*, *Bilophila*, and *Phascolarctobacterium* showed a significant increase in response to PAP1b, while *Klebsiella* showed a decrease in relative abundance. This change clearly indicated that PAP1b supplementation produced a significant modifying effect on the gut microbial community. Genomic analysis of *Lachnospiraceae*, an important component of the mammalian gastrointestinal microbiome, further confirmed their ability to efficiently utilize foodborne polysaccharides. Notably, several studies have reported that some *Lachnospiraceae* strains could metabolize saccharides, which in turn produce SCFAs such as butyrate, which play key roles in the regulation of the immune system [47]. The increase in the relative abundance of *Lachnospiraceae* resulting from the PAP1b intervention might imply that gut microbes could play a more active role in promoting immune system health. Further studies have also shown that the *Lachnospiraceae* family exhibit the ability to inhibit the proliferation of colorectal cancer cells, even in the complex microenvironment of gut contents [48]. *Lachnoclostridium*, an important bacterium, is a major producer of butyric acid, which in turn is a key source of nutrients for intestinal epithelial cells, further suggesting that PAP1b contributes to the enrichment of bacteria that are capable of producing SCFAs. *Phascolarctobacterium*, belonging to the *Firmicutes* phylum, possesses the ability to utilize succinate in order to produce propionic acid [49]. On the other hand, the PAP1b group reduced *Klebsiella* and a reduction in *Klebsiella* was beneficial for host health, as this bacterium is often associated with pneumonic sepsis in humans and may lead to high morbidity and mortality. In the INU group, elevated *Bifidobacterium* abundance was observed. *Bifidobacterium* has the ability to metabolize glycans, and this ability was positively correlated with gut health. In fact, Geng et al. [36] also came up with similar results in their inulin study. In conclusion, the addition of PAP1b not only contributed to the maintenance of intestinal health but also played an immunomodulatory role. By modifying the composition of gut microorganisms, specifically increasing the abundance of beneficial bacteria such as *Lachnospiraceae*, *Lachnoclostridium*, *Bilophila*, and *Phascolarctobacterium* while decreasing the number of potentially harmful bacteria such as *Klebsiella*, PAP1b could provide positive support for gut health and overall immune function.

**Figure 4:**
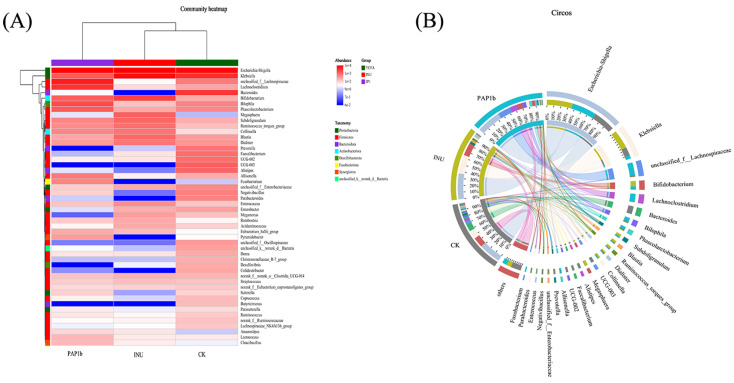
Species-level heat map of PAP1b; (**A**) fermentation and (**B**) Circos analysis.

As shown in Figure 4A,B, there was a significant distance between the bacterial community in the PAP1b group, the CK group, and the INU group, implying that they showed a large difference in species composition. The enrichment of *Lachnoclostridium* was particularly significant in the PAP1b group compared with the control group. *Lachnoclostridium*, as an important bacterium, is one of the main producers of butyric acid, an indispensable source of nutrients for intestinal epithelial cells [50]. This significant enrichment suggests that PAP1b could promote the growth of bacteria capable of producing SCFAs, which has positive implications for improving human intestinal metabolism. On the other hand, the abundance of *Megasphaera* was overwhelmingly dominant in the INU group compared with the CK group. It has been shown that *Megasphaera* are able to promote lactate and butyrate production in vivo [51]. In conclusion, PAP1b markedly altered gut microbiota composition, suggesting potential benefits for intestinal function and metabolic regulation. These findings could provide a basis for further studies to validate its prebiotic effects and underlying mechanisms in vivo.

**Figure 5:**
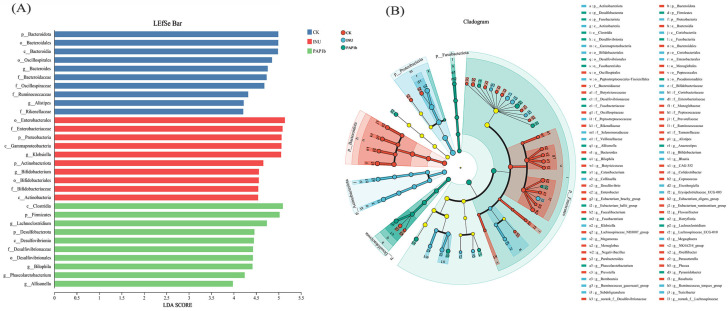
(**A**) LDA score and (**B**) Lefse analysis of gut microbiota. Colored areas represent groups with distinct microbial enrichment; colored nodes indicate differential taxa, while yellow nodes denote non-significant common ancestors. The outer labels match the group where each taxon is enriched.

Linear discriminant analysis (LDA) is a useful approach for assessing gut microbiota shifts under different interventions, with LDA scores indicating the magnitude of group differences. As presented in Figure 5A, the LDA scores fell within the range of 4 to 5.5, which clearly indicated that there were significant differences between different microbiomes. Significantly different taxa were identified and interpreted using LEfSe analysis (Figure 5B). *Clostridia* play an integral role in plant polysaccharide fermentation and utilization and SCFA production, as well as improving the body’s metabolism to enhance immunity. It has been demonstrated that *C. butyricum* has significant beneficial effects on antioxidant properties, growth performance, gut microbiota, and intestinal immunity [52]. *C. butyricum* is likely to be a potential alternative to antibiotics for the prevention of intestinal diseases. *Phylum Firmicutes* has many genes responsible for the fermentation of dietary fiber, which interact with the intestinal mucosa and may contribute to host homeostasis. These results highlight the role of gut microbiota in sustaining human health and offer insights for developing therapeutic approaches targeting obesity, metabolic disorders, and immune dysfunction. Notably, *Lachnoclostridium*, as the main beneficial gut microbiota involved in plant polysaccharide fermentation and utilization to produce SCFAs, plays an important role in improving body metabolism, reducing obesity, and enhancing immunity [53]. In addition, certain *Lachnoclostridium* strains might have preventive or therapeutic effects on diseases such as intestinal inflammation, obesity, and cardiovascular disease through modulating the immune system, inhibiting the growth of pathogens, or producing beneficial metabolites [50].

PAP1b could promote the production of butyric acid by *Clostridia*, which not only further lowers the intestinal pH but also inhibits the growth of pathogenic bacteria and sulfate-reducing bacteria, and at the same time could enhance the intestinal barrier function by activating the *PPAR-γ* signaling pathway of the host cells, providing a more stable environment for the survival of itself and other commensal bacteria. The monosaccharides or oligosaccharides generated from the decomposition of PAP1b by *Firmicutes* may be further metabolized by *Clostridia* to form a mutually beneficial symbiotic network. For example, the acetic acid produced by *Firmicutes* could be used as a precursor for the synthesis of butyric acid by *Clostridia*, and the low pH environment of butyric acid may inhibit the other competing flora and consolidate the dominant position of both [54]. PAP1b regulates the intestinal microenvironment by significantly up-regulating flora such as *Clostridia* and *Firmicutes*.

### 3.4. Impacts of PAP1b on Gut Fermentation Metabolites

#### 3.4.1. Metabolite Composition and Diversity

**Figure 6:**
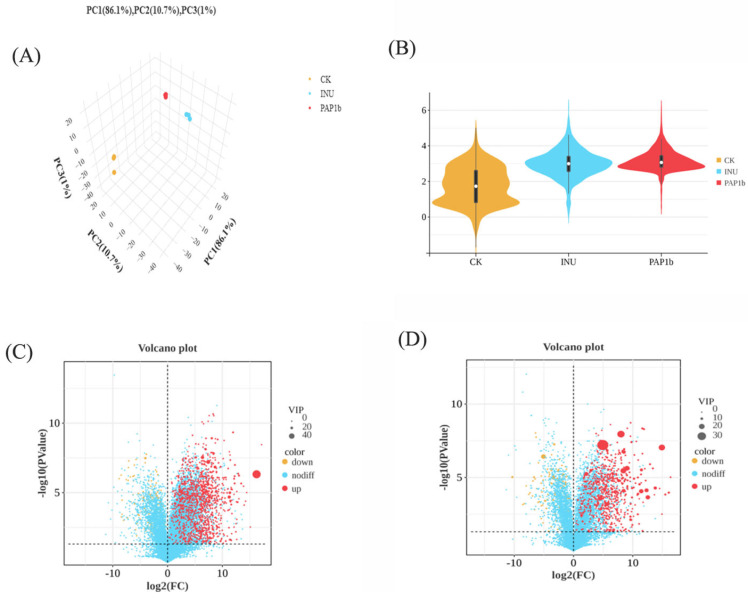
(**A**) Microbial diversity, (**B**) *β*-diversity, diagram of metabolite volcanoes between the two groups in anionic and cationic mode (**C**,**D**).

Metabolomics is a powerful approach for elucidating how microbial metabolites act as signaling molecules in host–microbiota interactions. To investigate the mechanisms by which PAP1b influences gut microbial communities, metabolites in the fermentation supernatant were profiled using UPLC-MS/MS, enabling a comprehensive assessment of metabolic changes associated with PAP1b treatment. The results showed that PAP1b had a significant effect on the metabolism of intestinal flora. The PCA score plot, in which PCA1, PCA2, and PCA3 accounted for 86.1%, 10.7%, and 1.0% of the variance, respectively (Figure 6A), showed a significant clustering of samples between the CK group and the PAP1b group, which suggests that PAP1b regulates the metabolism of the intestinal flora. In addition, the PAP1b group and the CK group were farther apart in the PCA plot, further indicating the regulatory effect of PAP1b on the metabolism of intestinal flora.

The PAP1b and the INU groups showed an extremely significant increase in the key index of intestinal flora abundance compared with other groups (Figure 6B). This finding not only profoundly revealed the positive association and differential characteristics between the samples but also further emphasized the unique value and important impact of the INU and PAP1b groups in the study of the field of intestinal microecology. Volcano plot analysis further revealed 1166 and 730 differentially abundant metabolites between the CK and PAP1b groups under negative ion mode (Figure 6C) and positive ion mode (Figure 6D), respectively, highlighting distinct metabolic profiles induced by PAP1b intervention. Among these significantly different metabolites in the anionic mode, 1018 were up-regulated and 148 were down-regulated.

Taken together, these results suggest that PAP1b could significantly affect the metabolism of the gut flora and acts by regulating the levels of multiple metabolites. This finding could provide a new perspective for understanding the interactions between gut microbes and host health, and a scientific basis for developing potential therapeutic approaches for health.

#### 3.4.2. Pathway Enrichment Analysis

**Figure 7:**
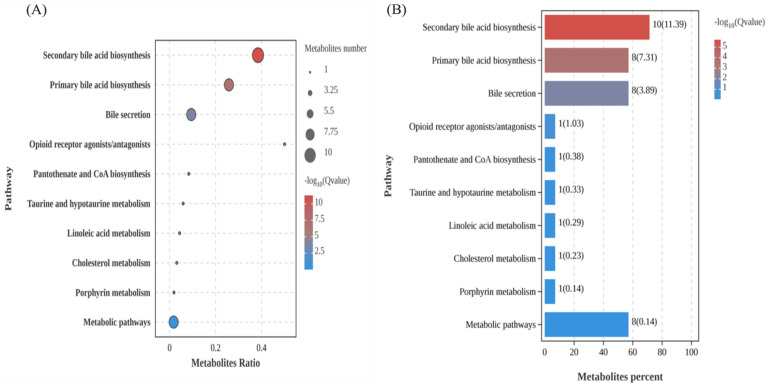
(**A**) KEGG metabolic pathway diagram and (**B**) enrichment bar diagram.

As illustrated in the Figure 7A,B, multiple metabolites were involved in several key biosynthetic and metabolic pathways: secondary bile acid biosynthesis (encompassing ursodeoxycholic acid, cholic acid, and taurocholic acid), primary bile acid biosynthesis (involving bile acids, taurocholic acid, and 5β-epoxy sulfate), bile secretory processes (encompassing bile acids, taurocholic acid, zydovidine, and cholestane-3,7,12,25-tetracosactyl-3-glucuronide), opioid receptor agonism, pantothenate and CoA biosynthesis (specifically panthenol), and porphyrin metabolism (involving steroidal cholagogues and sirohydrochlorin).

After intervention with PAP1b, the bile secretion and metabolism pathways showed significant enrichment. Bile secretion, as a crucial metabolic pathway, was significantly enriched by the intervention of PAP1b compared with the CK group in Figure 7A,B. In the PAP1b group, metabolites such as serotonin, deoxycholic acid, D-glucose, cortisol, phenylethylamine glucosinolate, and zalcitabine were involved in the bile secretion process (Table S1). Available evidence suggested that disturbances in bile secretion were strongly associated with the development of experimental colitis [55]. Bile was able to attenuate the inflammatory response by inhibiting the activation of inflammatory vesicles and promoting the proliferation and differentiation of intestinal stem cells, which in turn improved the intestinal barrier function in mice. In addition, there was a correlation between enhanced tryptophan metabolism and the reduction of DSS-induced colitis symptoms. Overall, derivatives of tryptophan metabolism may act as potential aryl hydrocarbon receptor (AHR) ligands to influence the progression of colitis by modulating immune responses [56]. PAP1b also might drive bile acid excretion into the intestine by activating the FXR receptor and enhancing bile acid synthase expression while promoting bile acid transporter protein function [57]. Our findings revealed that PAP1b might inhibit inflammation by promoting bile secretion while enhancing tryptophan metabolism to modulate immune responses, resulting in effective relief of colitis symptoms. These results are of clinical relevance, as dysbiosis of the gut microbiota and disruptions in amino acid metabolism have been linked to diverse pathologies, including metabolic disorders, neurodegenerative conditions, and gastrointestinal diseases. Understanding these associations may aid in developing targeted interventions to restore microbial balance and metabolic homeostasis [58].

### 3.5. Correlation Analysis of Gut Microbes and Metabolites

Combining microbial community and metabolomics analysis tools, PAP1b’s fermentation metabolites could effectively regulate the abundance of the thick-walled and anamorphic phyla and affect bile metabolic processes significantly. Correlation studies revealed that *C. luteus* polysaccharides (CLP) possessed preventive health functions and significantly influenced the gut microbial–bile acid (BA) axis dynamics associated with hyperlipidemia, mainly through elevating the abundance of *Lactobacillus paracasei* and the associated butyric acid concentration [59]. Oat polysaccharides may influence cardiovascular health by reducing cholesterol via bile acid metabolism, enhancing reverse cholesterol transport (RCT), promoting SCFA production, modulating bacterial cholesterol metabolism, and regulating microbe–host signaling pathways [60]. The enrichment of bile acid metabolism and carbohydrate utilization pathways in the PAP1b group aligns with patterns reported for jujube polysaccharides, indicating a shared mechanism by which plant-derived polysaccharides regulate host–microbiota interactions [8].

SCFAs serve as a key energy supply for intestinal epithelial cells and act as precursors for gluconeogenesis, contributing to the regulation of glucose homeostasis [61]. Different gut microbial fermentation activities could result in variable SCFA production. Propionate plays a central role in lipid metabolism due to its ability to effectively block fatty acid production in the in vitro environment, in contrast with acetate and butyrate, which are the main raw materials in the lipogenesis process. The elevated levels of propionate may be able to bring about a lipid-lowering effect, implying that PAP1b might possess lipid-lowering potential, a speculation supported by data from previous studies [62]. Butyric acid has been widely demonstrated to have multiple positive effects on intestinal health, including providing energy to intestinal cells, maintaining water and electrolyte balance in the body, regulating the structure of intestinal microbial communities, exerting anti-inflammatory effects, and influencing gene expression [63]. In addition, the production of isobutyric acid, valeric acid, and isovaleric acid in different groups, similar to the trend of acetic acid and propionic acid, all gradually could increase with the depth of the fermentation process, which could provide a reference value for exploring more potentials of AP.

In this research, the intervention of PAP1b significantly up-regulated the abundance of *Firmicutes* in the intestinal flora and inhibited the proliferation of *Bacteroidota*. The increasing abundance of *Firmicutes*, the main dietary fiber-fermenting bacterial group, directly promoted the production of SCFAs. In addition, acetic and propionic acids were involved in the regulation of host glycolipid metabolism and energy homeostasis by the activation of G protein-coupled receptors (e.g., GPCR43) in the intestines and liver [64]. Down-regulation of the *Bacteroidota* phylum might reduce its competitive utilization of host mucopolysaccharides, indirectly providing more substrates for fibrous fermentation by thick-walled bacteria and further enhancing the synthesis efficiency of SCFAs [65]. This alteration of the bacterial population structure suggests that PAP1b might promote the production of SCFAs by regulating the intestinal microecological balance, which in turn positively affects host metabolism and immune homeostasis. The up-regulation of bile acid metabolism might be another notable feature of PAP1b intervention, and its mechanism might be closely related to the interaction between the flora and its metabolites. Bile acids are produced from cholesterol in the liver and metabolized by intestinal flora in the intestine.

Conversely, bile acids might regulate the gut microbial composition directly or indirectly by activating innate immune genes in the small intestine [66]. In this experiment, SCFAs could reduce intestinal pH and inhibit the growth of some harmful bacteria, indirectly affecting the activity of bile acid-transforming bacteria (e.g., *Bacteroidota* phylum) and forming a metabolic feedback loop. PAP1b, as a fermentable fiber, enriches *Firmicutes* phylum, increases the production of SCFAs (especially butyric acid), strengthens the intestinal barrier, inhibits inflammatory responses, and regulates glycolipid metabolism in peripheral tissues [67]. SCFAs could affect bile acid-converting activity [68]; at the same time, bile acid signaling feedback regulates flora composition and SCFA production, forming a dynamically balanced metabolic loop. This bidirectional regulation suggests that flora and SCFAs are not only directly involved in bile acid biotransformation but also jointly regulate lipid uptake and energy homeostasis [57]. This integrative mechanism reveals that PAP1b activates SCFAs and bile acid metabolic pathways by remodeling the structure of intestinal flora, ultimately realizing the multilevel regulation of host metabolic homeostasis.

## 4. Conclusions

PAP1b underwent in vitro fermentation and led to a continuous reduction in pH and a marked increase in SCFAs levels. It significantly influenced the gut microbial composition, with a notable enrichment of *Firmicutes* and related genera. Metabolomic profiling revealed alterations in bile acid metabolism and indicated microbial shifts involving *Firmicutes*, *Bacteroidota*, and *Actinobacteriota*. These findings suggest that PAP1b might modulate gut microbial metabolism and related biochemical pathways under simulated gastrointestinal conditions. Further investigations using in vivo models and integrated omics approaches are required to confirm its functional relevance and underlying mechanisms.

## Data Availability

The original contributions presented in this study are included in the article/Supplementary Materials. Further inquiries could be directed to the corresponding authors.

## Supplementary Materials

The following supporting information could be downloaded at: https://www.mdpi.com/article/10.3390/foods14172954/s1, Table S1: Metabolites under positive and negative ions.

## References

[1] Peng W., Liu Y.J., Wu N., Sun T., He X.Y., Gao Y.X., Wu C.J. (2015). *Areca catechu* L. (Arecaceae): A review of its traditional uses, botany, phytochemistry, pharmacology and toxicology. J. Ethnopharmacol..

[2] Yin M.S., Pan F.B., Guo J.H., Ji X.L., Liu Y.Q. (2021). Research into chemical constituents and pharmacological activities in *Areca catechu* L.. Food Res. Dev..

[3] Zhang M.F., Zhuang H.N., Zhang X.W., Wang X.Y., Fu X., Chen S., Yao L.Y., Wang H.T., Sun M., Yu C. (2023). Structural characteristics of areca nut seed neutral polysaccharide and its impact on gut microbiota from human feces. Food Hydrocolloid..

[4] Ji X.L., Guo J.H., Pan F.B., Kuang F.J., Chen H.M., Guo X.D., Liu Y.Q. (2022). Structural elucidation and antioxidant activities of a neutral polysaccharide from arecanut (*Areca catechu* L.). Front. Nutr..

[5] Sun Y.J., Yao J.X., Gao R.N., Hao J.Y., Liu Y., Liu S. (2025). Interactions of non-starch polysaccharides with the gut microbiota and the effect of non-starch polysaccharides with different structures on the metabolism of the gut microbiota: A review. Int. J. Biol. Macromol..

[6] Zhang D.D., Liu J., Cheng H., Wang H.L., Tan Y.Z., Feng W.W., Peng C. (2022). Interactions between polysaccharides and gut microbiota: A metabolomic and microbial review. Food Res. Int..

[7] Guan X., Feng Y., Jiang Y., Hu Y., Zhang J., Li Z., Song C., Li F., Hou J., Shen T. (2022). Simulated digestion and in vitro fermentation of a polysaccharide from lotus (*Nelumbo nucifera* gaertn.) root residue by the human gut microbiota. Food Res. Int..

[8] Ji X.L., Chen J., Li Z.R., Meng Y., Li X.Q. (2024). Digestion characteristics of jujube polysaccharide and its regulatory effect on intestinal microbiota and metabolites during in vitro fermentation. LWT-Food Sci. Technol..

[9] Guo Y.X., Chen X.F., Gong P., Chen F.X., Cui D.D., Wang M.R. (2021). Advances in the in vitro digestion and fermentation of polysaccharides. Int. J. Food Sci. Technol..

[10] Ye S.X., Shah B.R., Li J., Liang H.S., Zhan F.C., Geng F., Li B. (2022). A critical review on interplay between dietary fibers and gut microbiota. Trends Food Sci. Technol..

[11] Yu C.X., Dong Q., Chen M.J., Zhao R.H., Zha L., Zhao Y., Zhang M.K., Zhang B.S., Ma A.M. (2023). The effect of mushroom dietary fiber on the gut microbiota and related health benefits: A review. J. Fungi.

[12] Hou C.Y., Chen L.L., Yang L.Z., Ji X.L. (2020). An insight into anti-inflammatory effects of natural polysaccharides. Int. J. Biol. Macromol..

[13] Xue H.K., Tang Y.Q., Zha M., Xie K.F., Tan J.Q. (2025). The structure-function relationships and interaction between polysaccharides and intestinal microbiota: A review. Int. J. Biol. Macromol..

[14] Ou J.Y., Wang Z., Liu X.F., Song B.B., Chen J.P., Li R., Jia X.J., Huang R.M., Xiang W.Z., Zhong S.Y. (2022). Regulatory effects of marine polysaccharides on gut microbiota dysbiosis: A review. Food Chem. X.

[15] Ma K.L., Kei N., Yang F., Lauw S., Chan P.L., Chen L., Cheung P.C.K. (2025). In vitro fermentation characteristics of fungal polysaccharides derived from *Wolfiporia cocos* and their effect on human fecal microbiota. Carbohyd. Polym..

[16] Lopes V.D., Brito F.O., Lopes J.D., Fabiano G.A., Lazarone C.S., Citelli M., Zago L., Antunes A.E.C., Evans M., Stenvinkel P. (2025). In vitro gastrointestinal digestion of broccolis sprout extract: Impact on the bioaccesibility of sulforaphane and total phenolic. Food Res. Int..

[17] Yan H., Fan C.J., Wang Y.J., Liu Z.L., Wang J.Q., Nie S.P. (2025). Structural characterization and in vitro fermentation of the polysaccharide from fruits of *Gardenia jasminoides*. Int. J. Biol. Macromol..

[18] Zhu K.X., Yao S.W., Zhang Y.J., Liu Q.B., Xu F., Wu G., Dong W.J., Tan L.H. (2019). Effects of in vitro saliva, gastric and intestinal digestion on the chemical properties, antioxidant activity of polysaccharide from *Artocarpus heterophyllus* Lam. (Jackfruit) Pulp. Food Hydrocolloid..

[19] Di T., Chen G., Sun Y., Ou S., Zeng X., Ye H. (2018). In vitro digestion by saliva, simulated gastric and small intestinal juices and fermentation by human fecal microbiota of sulfated polysaccharides from *Gracilaria rubra*. J. Funct. Foods.

[20] Lu J., You Z.S., Zhang Y.H., Wang F., Wang L.F., Xiong L., Song H.Z., Shen X.C. (2025). Structural characterization and in vitro fermentation properties of polysaccharides from *Dragon fruit* (*Hylocereus undatus*). J. Agric. Food Chem..

[21] Jiang C.P., Li H.Y., Li J.Q., Zou G.Y., Li C., Fang Z.F., Hu B., Wu W.J., Li X.L., Zeng Z. (2024). In vitro simulated digestion and fermentation behaviors of polysaccharides from *Pleurotus cornucopiae* and their impact on the gut microbiota. Food Funct..

[22] Xia C.M., Xu X., Zhang R.F., Su D.X., Jia X.C., Deng M., Lee Y.K., Zhang M.W., Huang F. (2025). Effects of molecular weight on simulated digestion and fecal fermentation of polysaccharides from longan pulp in vitro. Int. J. Biol. Macromol..

[23] Ji X.L., Hou C.Y., Yan Y.Z., Shi M.M., Liu Y.Q. (2020). Comparison of structural characterization and antioxidant activity of polysaccharides from jujube (*Ziziphus jujuba* Mill.) fruit. Int. J. Biol. Macromol..

[24] Liang X.F., Liu M.Q., Yao A.N., Cui W.C., Wei Y., Guo S., Duan J.L., Kang H.J., Zhou X.Y., Su S.L. (2024). In vitro fermentation characteristics and interaction of neutral and acidic polysaccharides from Lycii fructus on human gut microbiota. Food Hydrocolloid..

[25] Ji X.L., Hou C.Y., Gao Y.G., Xue Y.Q., Yan Y.Z., Guo X.D. (2020). Metagenomic analysis of gut microbiota modulatory effects of jujube (*Ziziphus jujuba* Mill.) polysaccharides in a colorectal cancer mouse model. Food Funct..

[26] Yang M., Cai W.H., Li X.X., Deng Y.X., Li J.J., Wang X., Zhu L.Y., Wang C., Li X.Q. (2024). The effect of type 2 resistant starch and indole-3-propionic acid on ameliorating high-fat-diet-induced hepatic steatosis and gut dysbiosis. Foods.

[27] Zhang S., Zhang M.X., Li W., Ma L.N., Liu X.L., Ding Q.T., Yu W.M., Yu T.J., Ding C.B., Liu W.C. (2023). Research progress of natural plant polysaccharides inhibiting inflammatory signaling pathways and regulating intestinal flora and metabolism to protect inflammatory bowel disease. Int. J. Biol. Macromol..

[28] Sun T., Liang X.N., Xu X.Y., Wang L.H., Xiao W., Ma Y.H., Wang R., Gu Y., Li S., Qiu Y.B. (2024). In vitro digestion and fecal fermentation of basidiospore-derived exopolysaccharides from *Naematelia aurantialba*. Int. J. Biol. Macromol..

[29] Zhang Y., Wang L.P., Qiu Z.H., Yang Y.T., Wang T.Z., Inam M., Ma H.X., Zhang H.P., He C.G., Guan L.L. (2024). Comprehensive evaluation of *Flammulina velutipes* residues polysaccharide based on in vitro digestion and human fecal fermentation. Int. J. Biol. Macromol..

[30] Fu Y.S., Zhang J.N., Chen K.N., Xiao C.X., Fan L.N., Zhang B.Z., Ren J.L., Fang B.S. (2019). An in vitro fermentation study on the effects of *Dendrobium officinale* polysaccharides on human intestinal microbiota from fecal microbiota transplantation donors. J. Funct. Foods.

[31] Wu D.T., Nie X.R., Gan R.Y., Guo H., Fu Y., Yuan Q., Zhang Q., Qin W. (2021). In vitro digestion and fecal fermentation behaviors of a pectic polysaccharide from okra (*Abelmoschus esculentus*) and its impacts on human gut microbiota. Food Hydrocolloid..

[32] Gao Y.Y., Guo Q.B., Zhang K.L., Wang N.F., Li C.R., Li Z.J., Zhang A.L., Wang C.L. (2020). Polysaccharide from *Pleurotus nebrodensis*: Physicochemical, structural characterization and in vitro fermentation characteristics. Int. J. Biol. Macromol..

[33] Wu D.T., An L.Y., Liu W., Hu Y.C., Wang S.P., Zou L. (2022). In vitro fecal fermentation properties of polysaccharides from *Tremella fuciformis* and related modulation effects on gut microbiota. Food Res. Int..

[34] Ringseis R., Gessner D.K., Eder K. (2020). The gut-liver axis in the control of energy metabolism and food intake in animals. Annu. Rev. Anim. Biosci..

[35] Zhang X., Aweya J.J., Huang Z.X., Kang Z.Y., Bai Z.H., Li K.H., He X.T., Liu Y., Chen X.Q., Cheong K.L. (2020). In vitro fermentation of *Gracilaria lemaneiformis* sulfated polysaccharides and its agaro-oligosaccharides by human fecal inocula and its impact on microbiota. Carbohyd. Polym..

[36] Geng X.R., Guo D.D., Bau T., Lei J.Y., Xu L.J., Cheng Y.F., Feng C.P., Meng J.L., Chang M.C. (2023). Effects of in vitro digestion and fecal fermentation on physico-chemical properties and metabolic behavior of polysaccharides from *Clitocybe squamulosa*. Food Chem. X.

[37] Zhao Y.Y., Aru V., Wang D., Wang P., Qin P.Y., Jiang Q.Q., Li Z.D., Engelsen S.B., Zhao X.Y. (2025). Deciphering the interplay between pectin structural variability, intestinal bioavailability and gut microbiota metabolism: A review. Carbohyd. Polym..

[38] Zhang W.Y., Hu B., Liu C., Hua H.Y., Guo Y.H., Cheng Y.L., Yao W.R., Qian H. (2022). Comprehensive analysis of *Sparassis crispa* polysaccharide characteristics during the in vitro digestion and fermentation model. Food Res. Int..

[39] Bai H.S., Liu T., Wang H.Y., Li Y.L., Wang Z.Z. (2025). Regulating effects of three polysaccharides on gut microbiota in felines and canines: Insights from in vitro digestion, fecal fermentation, and lactic acid bacterial fermentation. J. Agric. Food Chem..

[40] Ji X.L., Yan Y.Z., Hou C.Y., Shi M.M., Liu Y.Q. (2020). Structural characterization of a galacturonic acid-rich polysaccharide from *Ziziphus jujuba* cv. Muzao. Int. J. Biol. Macromol..

[41] Guo Y.X., Chen X.F., Gong P., Wang M.R., Yao W.B., Yang W.J., Chen F.X. (2022). In vitro digestion and fecal fermentation of *Siraitia grosvenorii* polysaccharide and its impact on human gut microbiota. Food Funct..

[42] Carbonero F., Benefiel A.C., Alizadeh-Ghamsari A.H., Gaskins H.R. (2012). Microbial pathways in colonic sulfur metabolism and links with health and disease. Front. Physiol..

[43] Li M., Su J., Wu J.H., Zhao D., Huang M.Q., Lu Y.P., Zheng J., Li H.H. (2024). The prebiotic activity of a novel polysaccharide extracted from Huangshui by fecal fermentation in vitro. Foods.

[44] Liu Y., Huang H.J., Fan J.T., Zhou H., Zhang Y.M., Cao Y.X., Jiang W., Zhang W., Deng J.M., Tan B.P. (2022). Effects of dietary non-starch polysaccharides level on the growth, intestinal flora and intestinal health of juvenile largemouth bass *Micropterus salmoides*. Aquaculture.

[45] Song Q.Q., Wang Y.K., Huang L.X., Shen M.Y., Yu Y., Yu Q., Chen Y., Xie J.H. (2021). Review of the relationships among polysaccharides, gut microbiota, and human health. Food Res. Int..

[46] Xu S.Y., Aweya J.J., Li N., Deng R.Y., Chen W.Y., Tang J., Cheong K. (2019). Microbial catabolism of *Porphyra haitanensis* polysaccharides by human gut microbiota. Food Chem..

[47] Gan Q.X., Chen L.L., Xian J.C., An G.Q., Wei H.B., Ma Y.T. (2024). Digestive characteristics of *Gastrodia elata* Blume polysaccharide and related impacts on human gut microbiota in vitro. J. Ethnopharmacol..

[48] Zhang H.X., Miyake T., Maekawa T., Mori H., Yasukawa D., Ohno M., Nishida A., Andoh A., Tani M. (2023). High abundance of Lachnospiraceae in the human gut microbiome is related to high immunoscores in advanced colorectal cancer. Cancer Immunol. Immun..

[49] Lecomte V., Kaakoush N.O., Maloney C.A., Raipuria M., Huinao K.D., Mitchell H.M., Morris M.J. (2015). Changes in gut microbiota in rats fed a high fat diet correlate with obesity-associated metabolic parameters. PLoS ONE.

[50] Yin D.F., Zhong Y.D., Liu H., Hu J.L. (2024). Lipid metabolism regulation by dietary polysaccharides with different structural properties. Int. J. Biol. Macromol..

[51] Hashizume K., Tsukahara T., Yamada K., Koyama H., Ushida K. (2003). *Megasphaera elsdenii* JCM1772T normalizes hyperlactate production in the large intestine of fructooligosaccharide-fed rats by stimulating butyrate production. J. Nutr..

[52] Cai K.Y., Chen J.Y., Zhang Z.P., Ye Y.W., Sang S.Y., Luo X.H., Wang Y.J., Shan K., Ou C.R., Jia L.L. (2025). Recent progress of *Clostridium butyricum* in fish culture: Maintenance of intestinal homeostasis, improvement of disease resistance, activation of immune signaling pathways, and positive effects on fish. Aquaculture.

[53] Cheng J.B., Hu J.L., Geng F., Nie S.P. (2022). Bacteroides utilization for dietary polysaccharides and their beneficial effects on gut health. Food Sci. Hum. Well..

[54] Xue H.K., Liang B.M., Wang Y., Gao H.Y., Fang S.S., Xie K.F., Tan J.Q. (2024). The regulatory effect of polysaccharides on the gut microbiota and their effect on human health: A review. Int. J. Biol. Macromol..

[55] Duboc H., Rajca S., Rainteau D., Benarous D., Maubert M.A., Quervain E., Thomas G., Barbu V., Humbert L., Despras G. (2013). Connecting dysbiosis, bile-acid dysmetabolism and gut inflammation in inflammatory bowel diseases. Gut.

[56] Yang W.Q., Ren D.Y., Zhao Y., Liu L., Yang X.B. (2021). Fuzhuan brick tea polysaccharide improved ulcerative colitis in association with gut microbiota-derived tryptophan metabolism. J. Agric. Food Chem..

[57] Sayin S.I., Wahlström A., Felin J., Jäntti S., Marschall H.U., Bamberg K., Angelin B., Hyötyläinen T., Oresic M., Bäckhed F. (2013). Gut microbiota regulates bile acid metabolism by reducing the levels of tauro-beta-muricholic acid, a naturally occurring FXR antagonist. Cell Metab..

[58] Zhang C., Yu L.L., Zhai Q.X., Zhao R.H., Zhao J.X., Zhang H., Chen W., Tian F.W. (2023). In vitro fermentation of heparin by the human gut microbiota: Changes in the microbiota community and metabolic functions. Food Chem..

[59] Long H.R., Huang R., Zhu S.J., Wang Z.H., Liu X.L., Zhu Z.J. (2015). Polysaccharide from *Caulerpa lentillifera* alleviates hyperlipidaemia through altering bile acid metabolism mediated by gut microbiota. Int. J. Biol. Macromol..

[60] Cheng W.Y., Lam K.L., Li X.J., Kong A.P.S., Cheung P.C.K. (2021). Circadian disruption-induced metabolic syndrome in mice is ameliorated by oat β-glucan mediated by gut microbiota. Carbohyd. Polym..

[61] Sonnenburg J.L., Bäckhed F. (2016). Diet-microbiota interactions as moderators of human metabolism. Nature.

[62] Wang X.M., Li W.D., Xiao L., Liu C.D., Qi H.M., Zhang Z.S. (2017). In vivo antihyperlipidemic and antioxidant activity of porphyran in hyperlipidemic mice. Carbohyd. Polym..

[63] Martin-Gallausiaux C., Marinelli L., Blottière H.M., Larraufie P., Lapaque N. (2021). SCFA: Mechanisms and functional importance in the gut. Proc. Nutr. Soc..

[64] Smith P.M., Howitt M.R., Panikov N., Michaud M., Gallini C.A., Bohlooly-Y M., Glickman J.N., Garrett W.S. (2013). The microbial metabolites, short-chain fatty acids, regulate colonic treg cell homeostasis. Science.

[65] Hansson G.C., Johansson M.E.V. (2008). The inner of the two Muc2 mucin-dependent mucus layers in colon is devoid of bacteria. Gut Microbes.

[66] Wahlström A., Sayin S.I., Marschall H.U., Bäckhed F. (2016). Intestinal crosstalk between bile acids and microbiota and its impact on host metabolism. Cell Metab..

[67] Kovatcheva-Datchary P., Nilsson A., Akrami R., Lee Y.S., De Vadder F., Arora T., Hallen A., Martens E., Björck I., Bäckhed F. (2015). Dietary fiber-induced improvement in glucose metabolism is associated with increased abundance of *Prevotella*. Cell Metab..

[68] Soret R., Chevalier J., De Coppet P., Poupeau G., Derkinderen P., Segain J.P., Neunlist M. (2010). Short-chain fatty acids regulate the enteric neurons and control gastrointestinal motility in rats. Gastroenterology.

